# Supplementary material for: Effects of El Niño/La Niña on the Number of Imported Shigellosis Cases in the Republic of Korea, 2004–2017
Source: Int J Environ Res Public Health. 2020 Dec 30;18(1):211. doi: 10.3390/ijerph18010211 (PMC7795629; doi:10.3390/ijerph18010211)
Supplement: Supplementary file 1 [file ijerph-18-00211-s001.pdf]

International Journal of
Environmental Research and Public Health

MDPI

Table S1. Sensitivity analysis results sorted in ascending order of the AIC*.

df † of ONI	df of ONI ‡ lag-month	df of DMI §	df of DMI lag-month	df of season	df of time trend	AIC
3	2	3	3	3	7	770.2
3	3	3	3	3	7	771.1
3	2	3	4	3	7	771.1
2	2	3	3	3	7	771.5
3	3	3	4	3	7	771.8
2	3	3	3	3	7	771.9
2	2	3	4	3	7	772.9
3	2	3	3	4	7	773.7
3	4	3	4	3	7	774.1
4	2	3	3	3	7	774.1
3	3	3	3	4	7	774.6
3	2	3	4	4	7	774.6
3	3	4	3	3	7	774.8
3	2	3	3	3	8	774.8
3	4	3	3	3	7	775.0
2	3	3	4	3	7	775.1
2	2	3	3	4	7	775.1
2	3	3	3	3	9	775.2
4	3	3	3	3	7	775.2
3	3	3	4	4	7	775.3
2	3	3	3	4	7	775.4
2	2	3	3	3	9	775.4
3	2	3	3	3	9	775.6
4	2	3	4	3	7	775.9
3	2	4	3	3	7	776.0
2	2	3	3	3	8	776.3
3	2	3	4	3	8	776.4
2	2	3	4	4	7	776.5
3	3	3	3	3	9	776.5
2	3	4	3	3	7	776.8
3	2	3	4	3	9	777.0
2	2	3	4	3	4	777.1
3	2	3	4	3	4	777.1
2	2	3	4	3	9	777.1
3	3	3	4	3	4	777.4

* Out of the total 2,187 combined cases, AICs of 35 cases were presented in ascending order.; †df: degree of freedom; ‡ ONI: Oceanic Niño Index; § DMI: Indian Ocean Dipole Mode Index.

Table S2. Number of outbound South Korean travellers distributed by travel destination.

Destination	2004-2010	2011-2017	2004-2017
Thailand	6,215,749	9,136,811	15,352,560
Philippines	3,943,697	8,720,200	12,663,897
Vietnam	2,754,738	7,930,498	10,685,236
Singapore	2,699,874	3,643,761	6,343,635
Cambodia	1,939,920	2,711,268	4,651,188
Indonesia	2,033,158	2,449,668	4,482,826
Malaysia	1,422,603	2,557,924	3,980,527
India	498,594	794,090	1,292,684
Laos	103,739	775,579	879,318
Myanmar	108,269	364,676	472,945
Maldives	131,315	211,809	343,124
Nepal	67,678	137,137	204,815
Sri Lanka	32,054	83,798	115,852
Bhutan	631	10,214	10,845
